# Classifications, Changes, and Challenges of Online Health Information Seekers during COVID-19

**DOI:** 10.3390/ijerph18189495

**Published:** 2021-09-09

**Authors:** Hanna Choi, Shinae Ahn

**Affiliations:** 1 Department of Nursing Science, Nambu University, Gwang-ju 62271, Korea; hanna.choi.kr@gmail.com; 2 Department of Nursing, Wonkwang University, Iksan 54538, Korea

**Keywords:** online, health information, eHealth, mHealth, consumer

## Abstract

Objectives: The purpose of this study was to explore consumers’ experiences before and during the COVID-19 outbreak to improve public health by providing effective consumer health information. Methods: Interviews were conducted with 20 health information consumers who were 18 or older until data saturation was reached. The selected participants were among users of the Korean National Health Insurance Service (NHIS). The data were collected before the COVID-19 outbreak (September 2014) and during the COVID-19 outbreak (October 2020) to describe experiences and changes before and during the pandemic. Data were analyzed according to the qualitative content analysis method. Results: As a result, 3 main domains and 10 subdomains were derived from classifications, changes, and challenges of online health information seekers. Conclusions: The findings of this study guide the understanding of health information seekers for the development of consumer-tailored health information systems.

## 1. Introduction

The proportion of general use of the internet in daily activities has continuously increased [1,2]. Online and mobile health apps have deeply intervened in daily life [3]. During the COVID-19 pandemic, consumers experienced far-reaching changes. People have stayed at home to follow physical distancing rules to prevent the spread of COVID-19. Due to prolonged stays at home with increased concerns about health-related issues, they have increasingly sought health information online [4]. Consumers visit online health sites and access information easily and then share it via YouTube, Facebook, Twitter, Google, and various online media [5,6]. Smartphone users have reported that they frequently use various types of applications, such as communication, photo and video, and health apps [2].

The need to provide health information that is easily accessible and helpful and that ensures the right to be informed has increased, which, subsequently, has enabled consumers to perform self-management more efficiently [7]. Consumers use the internet for various health-related activities, such as obtaining health information and learning about treatment options and health-promoting activities [8]. A meta-analysis reported that consumers are more involved in self-managed care in the early detection, prevention, and treatment of disease [9]. This has led to changes in the types of health care, depending on the health condition, from early detection and disease prevention to self-care in treating disorders [9,10].

Health information constantly changes with social trends, including the COVID-19 pandemic, such as developments in life sciences and information technology, requiring the modification of content or platforms to meet the recipients’ demands [11]. However, the current state of and changes in health information utilization and its influence on daily life are not specifically measured [12]. Thus, a national health information provider should be able to identify the current needs of consumers as well as trends and be able to reflect them immediately so that the correct provision of health information can contribute to promoting national health [13].

A variety of qualitative studies has examined motivation, health information searches, and evaluations of various age groups, genders, and diseases [7,14,15], using social media or mobile phones [16,17]. Other studies have qualitatively defined health information-seeking behaviors [8] and conducted a concept analysis [18]. These studies can measure factors, such as the consumers’ characteristics and their satisfaction with the homepage usage, as well as offer quantitative tools in providing health information. However, these studies have limitations in identifying the specific satisfaction or desires related to consumers’ changing health information-seeking experiences concerning the COVID-19 pandemic. There is a need for research that explores why, when, and where consumers obtain health information and the way health information is used in their health care management from the consumers’ point of view. Therefore, this study utilized qualitative research methods involving a focus group and individual interviews to describe the consumers’ experiences of using health information before and during the COVID-19 pandemic. The qualitative methods contribute to a deeper understanding of the participants’ experiences in different contexts and situations [19]. Thus, to explore consumers’ perspectives of obtaining health information and behavioral changes related to the use of health information in depth, the qualitative research design is suitable.

This study is a description focusing on the experiences of consumers to explore changes in online health information-seeking behaviors and self-health management. In this regard, we aim to present a vivid description by gaining an in-depth understanding of users’ online health information experiences in the last 6 years when faced with sporadic health information before and during the COVID-19 pandemic. Our study findings may contribute to the better provision of health information and help increase the efficiency of the use of health information not only by the users but also consumers and health-related stakeholders after the COVID-19 pandemic.

## 2. Materials and Methods

### 2.1. Study Design

This research is a qualitative study using content analysis of the experiences of consumers who explored online health information via a focus group and individual interviews.

### 2.2. Study Participants

To construct the focus group, 15 consumer volunteers out of 10,149 who participated in an online questionnaire from September 15 to 19, 2014, for a qualitative NHIS investigation were selected using the random selection method in Excel. Then, eight individuals who voluntarily agreed to participate in the research via phone formed the primary focus group members. After 6 years, secondary individual interviews were conducted with 3 of the original participants as well as 12 new participants during the COVID-19 pandemic. According to the focus group research method [20,21], the suggested size for one focus group is 6–10 participants. In this study, eight participants were included in one group.

### 2.3. Recruitment of Research Participants and Data Collection

This research was approved by the institutional review board of Seoul National University (SNUIRB No. E1405/001-011), Nambu University (IRB No.1041478-2018-HR-007). For the recruitment of study participants, among 10,149 consumers who completed an online qualitative investigation survey, we randomly selected 15 consumers, who agreed to participate in an interview. Of the 15 participants, 8 voluntarily agreed to participate in the study in 2014. After 6 years, 12 participants who were users of Korea’s National Health Insurance Services and 3 participants who completed the previous FGI participated in the interviews in 2020. After the recruitment of participants, interviews continued until data saturation was reached, that is, until no new data, themes, or information emerged in the interviews [22].

On the day before the interview date, documents explaining the focus group method were sent via email. When the participants arrived at the promised time and location, they received research consent and consultation fee forms. In this process, we explained the purpose of the research, the guarantee of privacy, and the participants’ ability to terminate their participation at any time if they no longer wanted to participate. The first FGI was conducted on 25 September 2014 in a lecture hall at Seoul National University School of Nursing with one moderator and one co-moderator. The second FGI was conducted on 19 October 2020 in a quiet space near Nambu University with one moderator. The moderator attempted to return participants to the topic if they strayed from it for too long. The FGI was conducted for about 2.5 h until no new topics were extracted. Verbal statements were recorded with the participants’ agreement. Additionally, the interviews were concluded by informing the participants that there could be a second attempt at data collection. To help with generalization of saturated data from the preliminary organization of the collected data, participants were individually contacted, and data were collected once more via a face-to-face meeting, phone call, or email. This took 122 h. Research participants received approximately 50 USD in compensation.

### 2.4. Data Analysis

The recorded content of the first interview was transcribed for data analysis. All researchers involved checked for additional content to add to the organized content to cover factors other than the participants’ spoken words (e.g., the atmosphere in the room). Transcription took approximately 10 h, and the transcribed interview content was organized using unique individual codes rather than names so that participants were unidentified [23]. The research questions were based on the technology acceptance model; the ways users come to accept and use a technology, including the background behind a consumer’s experience and why, when, where, and how health information was explored; and how health information should be explored in the future (Table 1). This was also linked to the fact that qualitative content analysis is dynamic rather than structured in a mold [24]. The transcribed data were analyzed using qualitative content analysis methods [19,25] for the extraction of major categories (Table 2).

### 2.5. Verifying the Validity of the Research Results

We utilized four standards (i.e., credibility, auditability, fittingness, and confirmability) to evaluate the validity of the qualitative data as suggested by Sandelowski [26]. To ensure the credibility of the data, participants were selected using the random sampling method in the first FGI and the convenient sampling method in the second FGI. We obtained insight from researchers who have experience with nation-level health information about whether our analysis content was skewed, and related reports and documents were considered. For auditability, we kept a detailed record of all steps ranging from the planning of the research to the description of the results after the research as well as participants’ actual statements. For fittingness and confirmability, the content was refined by obtaining the expert opinions of two professionals who have proficient experience in qualitative research and two professors who are consumer-health informatics professionals, as well as the medical informatics department, on whether the transcription was appropriately summarized and whether the content contained ambiguity. To test the validity of the extracted topics, all participants were first asked in 2014 how much the described topics and the fundamentals of the experiences matched up, followed by reconfirmation of the topics in 2020 by the research participants and the co-moderator.

## 3. Results

The general characteristics of the 20 participants are presented in Table 3. Eight interviewees (40%) were male and twelve were female (60%). The average age was 42.9 (SD = 11.15), and 80% had graduated from university.

By analyzing the data using qualitative content analysis methods, the online health information seekers’ classifications, changes, and challenges were extracted as well as 10 subareas (Table 4).

### 3.1. Classifications

#### 3.1.1. Response When Problems Occur

People who search for information when problems occur normally do not care about and do not periodically look for health information, but they search for it when they are sick or need help. People in this category visit websites or apps to obtain information whenever they have a one-time curiosity, which enables them to learn health communication skills to help them communicate accurately with the medical team. Thus, they belong in the subareas of discovery of disease, recognition of symptoms, self-diagnosis of disease name, and search for medical institution in the course of active medical care and health.

#### 3.1.2. Prevention and Management

People in the preventative group search for information that is easy to follow, such as simple exercises that can be done at home or in the office. Their search behavior is more related to living a healthy lifestyle, such as stretching, rather than diseases. In addition, this refers to the type of management that is applied in real life by using videos or search results when maintenance of continuous management is necessary due to the chronic nature of a condition rather than ending with treatment in a short period.

#### 3.1.3. Health Information Exploration

People who explore health information tend to search the internet and click on health-related articles and videos to obtain instant information. They tend to read an article if it seems interesting or even if it has a minimal relationship to them. People in this category conduct a search even if they do not have symptoms. This category belongs in the promotion of health and prevention of disease in the health journey.

#### 3.1.4. Educator

People in the educator category search for health information to solve health problems for other people (e.g., a spouse, child, or parent) because they are the protectors of family health or because health education is their occupation. This group has considerable knowledge and information in practice. However, this group receives help gradually to make application of the information easier. This group belongs in the consultation participation group in the health journey subarea.

### 3.2. Changes

#### 3.2.1. Beyond the Pandemic: Increased Interest on Health

Before the COVID-19 pandemic, consumers did not conduct health-related searches often on the internet, except when they or their relatives were sick. However, during the COVID-19 pandemic, consumers conducted health-related searches more frequently. In particular, as time spent outside decreases and time at home increases, consumers find many health-related activities, such as dieting and home workouts, can be carried out in the time spent at home. In addition, they search for easily explained information about health, including foods and exercise to boost immunity. With the growing interest in infectious diseases, people became interested in detailed information, such as the mechanism of infectious disease, prognosis, disease incidence, and epidemiological investigations, beyond simple health information.

“I haven’t done health-related searches on the Internet except when I’m sick, but after COVID-19, I think I’ve been doing health-related searches more often. Even if my health deteriorated even a little, I started to look for more about the symptoms of COVID-19 and dietary supplements (ex. vitamins, calcium, magnesium, lutein, omega-3, etc.).”

#### 3.2.2. Prefer YouTube Videos to Online Articles

In the past 5–6 years, consumers often used SNS or websites through computers or mobile devices by visiting websites and apps, such as Facebook and Instagram. However, within the last 1–2 years, consumers mostly sought information through short videos, such as those found on YouTube. Rather than reading health-related articles, they check the news related to COVID-19 through videos and check the changing regional and international COVID-19 incidence rates.

“When I watch some content, YouTube recommends similar content to appear at the top, so it seems that I continue to watch related content.”

#### 3.2.3. Click on eHealth Content

Government-issued disaster notifications given through text alarms, which were previously turned off, are turned on nowadays. As the number of confirmed cases came in through text messages every day, when the alarms went off, consumers said they searched for articles related to COVID-19 news. While seeking health information, consumers checked the number of confirmed new COVID-19 cases per day in the area where they or their family members lived. They also found an interest in the social impact caused by COVID-19, vaccines, and even secondary sequelae.

“There was a time when I had a fever and severe muscle pain, so I was looking for health information because I was afraid it was COVID-19.”

### 3.3. Challenges

#### 3.3.1. Linking to Health Insurance Companies

To be covered by affordable health insurance, one must have medical certification to prove it. However, some of the data are stored in the HealthiN database as information only for hospital consultations. Under the principle of confidentiality, even if a patient agrees to link companies, other laws need to be enacted. Furthermore, this process is long and complicated, resulting in numerous consumers having a hard time obtaining affordable health insurance.

“If my personal information is on the site, do I have to claim insurance by getting all the certifications myself? It’ll probably be a lot more convenient for us if we just let the insurance company do it on the website.”

#### 3.3.2. Synchronizing Personal Data from Health Exams and Wearable Devices

Health information is not generated in one place but rather through various ways in several hospitals. Consumers hope that technologies and policies that bring these resources together are based on user consent. Departments focusing on regular checkups or treatments represent different parts of a hospital. Therefore, the data from different resources are scattered. These days, consumers use wearable devices that generate various health-related data, such as heart rate, steps, and sleep-related data. This information is used as good health-related data, but it would be convenient if the information could be connected to health management.

“I wish my data, which is scattered across different hospitals and smartwatches, could be gathered in one place.”

#### 3.3.3. Infodemics

The reliability of health information has been raised continuously. With the many opinions and various information provided online, the public feels that articles have a limited ability to help. Users are confused by information that differs according to who first drafted it (e.g., a doctor, journalist, or trainer) or that comes from unknown sources. When providing health-related information online, consumers pointed out that efforts are necessary to increase the credibility of the information, such as the introduction of a certification system that requires the source to be accurately indicated.

“When it became known that the code of conduct, I was trying to follow was wrong, I was embarrassed. It would be nice if you could filter out such misinformation.”

## 4. Discussion

Understanding consumers’ experiences in depth through qualitative research methods is essential to identify the factors that facilitate or hamper the search for health information and to establish a support system for consumers’ self-management competency using health information. This research is significant in that it examines people’s experiences using health information, centering on the NHIS from the users’ perspective. Through FGI and individual interview methods, users’ characteristics and demands were collected by listening to their experiences with seeking health information. Finally, we qualitatively analyzed the collected data secondarily concerning how the quickly changing web environment, consumer experiences, and demands had evolved after 6 years had passed. The results showed that the users’ demands had not changed drastically in 6 years. Therefore, more effort is required to identify in detail consumers’ needs in response to changing circumstances and to reflect them in the generation and use of health information.

Health care consumers can largely be categorized into three types [27]. In previous studies, they were categorized into “traditional health worry type”, “health information power user type”, and “health information exploration type” [27]. The purpose of categorizing consumers by type is that different demands and use behaviors are seen depending on the consumers’ type in searching for and choosing health information using the internet. We found that health information-seeking behaviors differ depending on the consumers’ type, and we were able to classify their desires. Thus, a multidimensional understanding of consumers’ health information-seeking behavior through specific classification is necessary.

The four types of health care consumer are widely distributed on Travis’s [28] health continuum. A consumer’s condition can potentially change from maximum stability to severe illness on the health continuum very rapidly. Therefore, it is necessary to place health information so that it is easy to access depending on the health journey and type to which the consumer belongs from the consumer’s perspective.

Recently, social media has become a part of consumers’ lives. Due to handheld mobiles and tablets, consumers can read various information. The technology supports medical information, requires a high level of literacy, and helps one to access medical information easily. The best-known social media platform is YouTube [29]. More than 2.3 billion consumers access the site once a month, with a 30.4% increase in users each year [30]. Following these dynamics, the study findings also reflect that YouTube worked as an important vehicle for sharing and disseminating timely health-related information and education tools [31].

Furthermore, concerns have existed about the quality of health information, which can be uploaded on the website by whoever wants to do so without sufficient regulations or guidelines. For instance, regarding the debate on COVID-19 vaccination side effects, biased information can mislead consumers [32]. To prevent the rapid spread of excessive unreliable information, slow the swift spread of misinformation, and make and share reliable information on the web, health care providers and the public require regulations, such as the HON code, which implies qualified health information is needed in the process of communicating health information [33].

Participants desired individualized and personalized services to help each person recognize his or her vulnerability and conditions that need management using the accumulated big data in one place. They also hoped that the data collected by the NHIS could be linked to health insurance directly and to simplify the process for health insurance billing. Issues will arise concerning infection-related health resources, policy-related areas, and sensitive areas of individual health information protection. However, participants emphasized that people would receive more benefits if individualized and personalized services can be considered under the agreement of the individual in the future.

Wearable devices, including health monitors, fitness bands, and smartwatches, provide a wide variety of data. The devices collect personal behavioral and physiological data that reflect consumers’ health statuses and activity levels from device sensors [34]. The benefits of wearable devices include managing lifestyle and breaking bad habits through haptic feedback to provide individual reports about health, behavior, and so forth. However, the functions exist within the device. Through the investigation of privacy and information security issues, if wearable devices synchronize personal health records, then they would increase patients’ opportunities to participate in and be responsible for their health care. In the context of health information seeking, patients can obtain health information through search engines and ask about disease or treatment through conversations with friends, physicians, and so forth [35]. As for health information seekers, by using wearable devices, data can support the clinician–consumer relationship and adjust new dynamics.

The patient engagement framework as defined by the National eHealth Collaborative has five stages: (a) “inform me”, (b) “engage me”, (c) “empower me”, (d) “partner with me”, and (e) “support my e-community” [36]. In this study, participants belonged in the first stage (i.e., inform me). To enhance personalized functions, it is necessary to expand the content that fits this stage and help consumers strengthen and expand to the highest stage (i.e., support my e-community) [34] because consumers can interact and engage in multiple environments in multiple ways. During the COVID-19 pandemic, eHealth technologies can be particularly effective resources to foster the health consumers’ active role in their health care process by providing reliable health information based on their needs. Previous research has shown that eHealth technologies using mobile applications and web portals directly improved patient’s self-management [37]. To improve patient engagement and their self-management, the study findings show that developing supportive or educational systems using health information technology according to consumers’ health conditions and level of health-information technology efficacy can be helpful.

This study has several limitations. First, though we analyzed the qualitative data until achieving data saturation, generalization of the findings requires caution because the participants were not representative of all consumers. Second, only general characteristics, such as gender, age, and education level, were included in this study. Thus, consumers’ personal and health-related characteristics may not be reflected. More demographic data, anthropometry, and psychological and physical conditions need to be included to compare subgroup differences within focus groups. Therefore, future research is suggested to explore consumers’ experiences stemming from online health information-seeking behaviors and changes in their self-management through a systematic sampling design with diverse characteristics of consumers.

## 5. Conclusions

This research was a qualitative study that examined health information-seeking experiences using a focus group and individual interviews of users who access online health information. The first interview of eight participants was conducted in 2014, before the COVID-19 pandemic, and three participants were asked about their current desires again through individualized interviews. During the COVID-19 pandemic in 2020, 12 participants had their first interview and 3 participants had their second interview after 6 years had passed. It was shown that changes do not automatically come with the passage of time; rather, more effort is required to decrease the gap between the rate at which consumers change and the developers’ content and services generation. Therefore, we aimed to refresh our understanding of the considerations and importance of the information that users (i.e., consumers) fundamentally want beyond information technology or changes in the user interface. Through continuous research, user experiences can be examined and help can be provided so that users can actively manage their health using online information. Our study suggests that a supportive system should be developed that reflects consumers’ needs and guides them to reliable health information. The findings from our study may be referenced for developing a supportive or educational system for consumers.

## Figures and Tables

**Table 1:** Guiding question topics from the focus groups.

Type	Question Topics
Introduction	· First experience finding health information (why, when, what, where, and how) · The main health information-related websites or applications participants used
Opening	· Opportunities to self-manage their health using the health information portal · Most memorable experience with OHISB · Change: OHISB in COVID-19 (second interview)
Exploratory	· Perceived usefulness: Whether OHISB improved their understanding of symptoms, diseases, treatments, and health care · How OHISB has affected their health planning and lifestyle (eating habits, exercise, etc.) · Perceived ease of use: Whether finding health information and managing health using online information saved time and effort as compared to using other forms of media · Inconvenient points in using online health information
Closing	· Attitude: Overall satisfaction with using the website or app to find health information and manage health · Behavioral intention: Whether they will continue to use the website or app for health management · Wishes: What content or technical support should be included when using online health information

OHISB = online health information-seeking behavior.

**Table 2:** Qualitative content analysis process.

Stage	Process	Role
1	Decontextualization	Identify unit of meaning Inductive or deductive coding system
2	Recontextualization	Compare with the original data Include content, exclude ‘dross’
3	Categorization	Bring subjects together Identify categories and subcategories
4	Compilation	Draw realistic conclusions

**Table 3:** General characteristics of participants. (*n* = 20).

Number	Gender	Age	Education	1st Phase (pre COVID-19)	2nd Phase (during COVID-19)
1	F	29	Bachelor’s degree	O	
2	M	43	Master’s degree	O	
3	F	50	Bachelor’s degree	O	
4	F	37	Bachelor’s degree	O	
5	F	38	Doctorate degree	O	
6	F	41	Bachelor’s degree	O	O
7	F	47	Bachelor’s degree	O	O
8	F	53	Master’s degree	O	O
9	M	59	High School Graduate		O
10	F	38	Bachelor’s degree		O
11	F	58	Master’s degree		O
12	F	25	High School Graduate		O
13	M	55	Doctorate degree		O
14	M	26	High School Graduate		O
15	M	26	High School Graduate		O
16	M	41	Doctorate degree		O
17	F	38	Master’s degree		O
18	F	53	Doctorate degree		O
19	M	59	Bachelor’s degree		O
20	M	42	Master’s degree		O

**Table 4:** Content analysis on participants’ experiences of the online health information seekers.

Domain	Sub-Domain	Quote
Classification	Response when problems occur	*One-time question-solving*
	Prevention and management	*Continuous use of health-related information such as on exercise and nutrition*
	Health information exploration	*Search when interested, even if the information is unrelated*
	Educator	*Seeking with a specific purpose*
Changes	Beyond the pandemic; Increased interest on health	*I think I’ve been doing health-related searches more often. Even if my health deteriorated even a little, I started to look for more about the symptoms of COVID-19 and dietary supplements.*
	Prefer YouTube videos to online articles	*When I watch content, YouTube recommends similar content, which appears at the top of the website, so it seems that I continue to watch related content.*
	Click on eHealth content	*There was a time when I had a fever and severe muscle pain, so I looked for health information because I was afraid it was COVID-19.*
Challenges	Linking to health insurance companies	*If my personal information is on the site, … [then] it’ll probably be a lot more convenient for us if we just let the insurance company do it on the website.*
	Synchronizing personal data from health exams and wearable devices	*I wish my data, which is scattered across different hospitals and smartwatches, could be gathered in one place.*
	Infodemics	*When it became known that the code of conduct, I was trying to follow was wrong, I was embarrassed. It would be nice if you could filter out such misinformation.*

## Data Availability

Data sharing is not applicable to this article.
